# Gut-derived signals regulating glial activation and secondary neuroinflammation after spinal cord injury: an evidence mapping and mechanistic framework

**DOI:** 10.3389/fncel.2026.1897379

**Published:** 2026-07-24

**Authors:** Baoxiu Yi, Wenguang Chen, Zhenhai Chi, Qiangjian Mao, Xiaoqin Li, Yuelai Chen

**Affiliations:** 1 College of Clinical Medicine, Jiangxi University of Traditional Chinese Medicine, Nanchang, Jiangxi, China; 2 The Affiliated Hospital of Jiangxi University of Chinese Medicine, Nanchang, Jiangxi, China; 3 Jiangxi Hospital Affiliated to Longhua Hospital, Shanghai University of Traditional Chinese Medicine, Nanchang, Jiangxi, China

**Keywords:** glial cells, gut-derived signals, gut–spinal cord axis, NLRP3 inflammasome, secondary neuroinflammation, spinal cord injury

## Abstract

Secondary neuroinflammation after spinal cord injury (SCI) is a key pathological process that affects neuronal survival, axonal regeneration, and functional recovery. Increasing evidence suggests that dysbiosis of the gut microbiota, disruption of the intestinal barrier, and abnormal microbial inflammatory and metabolic signals may promote the progression of secondary injury after SCI. However, direct, continuous, and cell-type-specific evidence explaining how gut-derived signals influence glial and neurovascular unit responses within the injured spinal cord through peripheral immune imbalance, blood-spinal cord barrier (BSCB) disruption, and local molecular pathways remains limited. In this narrative review, we organize the existing literature into an evidence map and propose a mechanistic hypothesis: After SCI, autonomic dysfunction, impaired gut motility, and neurogenic bowel dysfunction may disrupt the homeostasis of gut microbiota and barrier, leading to lipopolysaccharide (LPS) overflow, reduced short-chain fatty acids (SCFAs), altered tryptophan metabolism, and increased trimethylamine N-oxide (TMAO). These signals may modulate the responses of microglia/infiltrating macrophages, astrocytes, and the neurovascular unit via peripheral immunity, BSCB, and pathways, including TLR4/NF-κB, NLRP3, and AhR. We also distinguish direct SCI evidence, single-study support, and extrapolated evidence, and specifically avoid presenting the tryptophan metabolite-AhR axis or TMAO-NLRP3 axis as established SCI pathways. Overall, the gut-spinal cord axis may provide a useful framework for understanding and targeting secondary neuroinflammation after SCI. Still, its causal chain, temporal characteristics, and cell-specific effects require further validation.

## 1 Introduction

The pathological process of SCI includes primary mechanical injury and secondary injury. Following primary injury, local ischemia and hypoxia, BSCB disruption, immune-cell infiltration, and cytokine release occur in the injured tissue, collectively driving the progression of secondary injury (Ahuja et al., 2017; Zhao et al., 2022). Neuroinflammation permeates the entire injury and repair process and regulates neuronal survival, myelin repair, axonal regeneration, and glial scar formation (Elmalky et al., 2024; Shafqat et al., 2023). A moderate inflammatory response helps clear necrotic tissue and initiate repair, while an excessive or prolonged activation leads to the accumulation of pro-inflammatory mediators and neurotoxic factors, aggravating secondary injury (Nascimento et al., 2025; Pang et al., 2021). The gut microbiota can influence host homeostasis and the progression of central nervous system (CNS) diseases through immune, metabolic, neural, and endocrine pathways (Nakhal et al., 2024; Cryan et al., 2019). Under physiological conditions, the intestinal barrier, mucosal immunity, and microbial signals like SCFAs and indole-derived tryptophan metabolites contribute to maintaining epithelial barrier function, peripheral immune tolerance, and glial homeostasis in the CNS (Erny et al., 2015; Parada Venegas et al., 2019; Ranhotra, 2024; Kim et al., 2013).

After SCI, intestinal homeostasis is disrupted. Autonomic dysfunction, reduced intestinal peristalsis, altered bowel rhythm, and neurogenic bowel dysfunction change the luminal environment and induce gut microbial dysbiosis. Antibiotic exposure, dietary changes, prolonged bed rest, and hospitalization may further exacerbate this dysbiosis (O’Connor et al., 2018). Clinical and animal studies suggest that the diversity, composition, and metabolic function of gut microbiota may change after SCI and may be related to the severity of the injury, disease stage, and functional recovery (Kigerl et al., 2016). Therefore, intestinal barrier injury, overflow of LPS and other pro-inflammatory signals, reduced SCFAs, and altered tryptophan metabolism may affect microglia, astrocytes, infiltrating macrophages, and neurovascular unit cells in the injured area through peripheral immune imbalance and BSCB disruption, leading to persistent secondary neuroinflammation after SCI (Dong et al., 2025a; Jing et al., 2023).

The current evidence strongly supports the occurrence of gut microbial dysbiosis after SCI, but most studies have focused on microbial composition, intestinal barrier disruption, systemic inflammation, and microbiota-targeted interventions. How gut-derived signals further influence local cellular responses in the injured spinal cord, particularly glial-cell-mediated secondary neuroinflammation, has not been systematically organized (Kigerl et al., 2018; Bazzocchi et al., 2021). The persistent neuroinflammation after SCI should not be viewed solely as a result of increased peripheral cytokines. It may also reflect the remodeling of microglia, astrocytes, infiltrating macrophages, and cells of the neurovascular unit after gut-derived inflammatory and metabolic signals are transmitted through peripheral immune imbalance, BSCB disruption, and local molecular pathways (Jin et al., 2021).

Compared to recent reviews by Dong et al., Chen et al. (2025), and Pagán-Rivera et al., this review does not merely revisit the composition of the microbiota or the effects of microbiota-directed interventions after SCI. Instead, it reorganizes the evidence of the gut-spinal cord axis along a continuous chain of gut-derived signals, molecular pathways, glial responses, and secondary neuroinflammation. Specifically, we consider LPS overflow, reduced SCFAs, altered tryptophan metabolism, and elevated TMAO as representative gut-derived signals and analyze how they influence the responses of myeloid cells, astrocytes, and neurovascular units through peripheral immune imbalance, BSCB disruption, and pathways such as TLR4/NF-κB, NLRP3, and AhR, ultimately contributing to the development and maintenance of secondary neuroinflammation.

Based on this framework, the review addresses three questions. First, which gut-derived inflammatory or metabolic signals are most likely to be involved in secondary neuroinflammation after SCI? Second, can these signals influence microglia/infiltrating macrophages, astrocytes, and neurovascular unit cells through peripheral immune imbalance, BSCB disruption, and pathways such as TLR4/NF-κB, NLRP3, and AhR? Third, which lines of evidence directly stem from SCI models, and which are extrapolated from other CNS diseases, intestinal inflammation, or metabolic inflammation? This article aims to propose a cautious mechanistic framework and identify key breakpoints in the evidence chain that require further validation, rather than presenting established cell-specific mechanisms.

**Figure 1:**
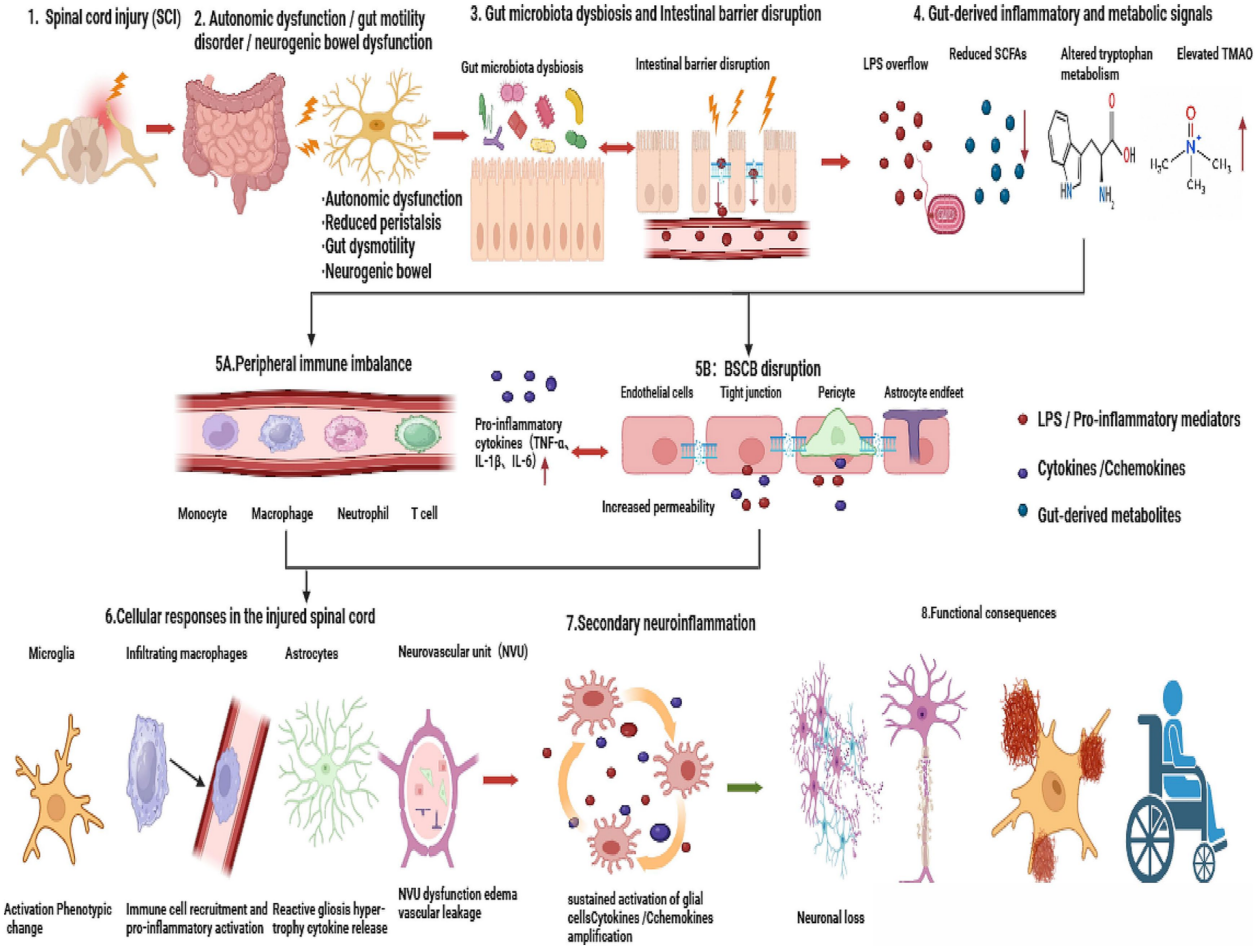
Overall mechanism of gut-spinal cord axis-mediated secondary neuroinflammation after SCI. After SCI, autonomic dysfunction, impaired gut motility, and neurogenic bowel dysfunction can disrupt gut microbiota and intestinal barrier function, resulting in LPS overflow, reduced SCFAs, altered tryptophan metabolism, and increased TMAO. These changes may induce peripheral immune imbalance and exacerbate BSCB disruption, thereby affecting microglia, infiltrating macrophages, astrocytes, and neurovascular unit responses and contributing to persistent secondary neuroinflammation and functional impairment.

This article is a narrative review rather than a systematic review or meta-analysis. The literature selection revolves around the following themes: gut microbiota dysbiosis after SCI, intestinal barrier disruption, gut-derived inflammatory and metabolic signals, BSCB injury, glial cell responses, and secondary neuroinflammation. Searches were conducted in PubMed, Web of Science, and Google Scholar using terms, including SCI, gut microbiota, gut-spinal cord axis, intestinal barrier, LPS, SCFAs, tryptophan metabolism, AhR, TMAO, NLRP3 inflammasome, microglia, macrophage, astrocyte, BSCB, and neuroinflammation. Recent animal experiments, clinical observational studies, and mechanistic studies related to SCI were prioritized. When direct evidence of SCI was insufficient, relevant evidence from other CNS diseases, intestinal inflammation, metabolic inflammation, and *in vitro* models was considered and explicitly identified in the text and tables as inferred evidence (Figure 1).

## 2 Gut microbiota dysbiosis and barrier dysfunction after SCI

The SCI disrupts sympathetic and parasympathetic regulation of gastrointestinal function, leading to reduced gut motility, prolonged intestinal transit time, altered bowel rhythm, abnormal mucus secretion, and changes in the luminal microenvironment. These changes, by affecting microbial colonization, substrate availability, and microbial metabolism, result in gut microbiota dysbiosis and barrier dysfunction, promoting the entry of luminal microbes and their metabolites into the circulatory system (Willits et al., 2024; Hamilton et al., 2024). In this sense, the intestinal changes after SCI are not limited to gastrointestinal complications. Through abnormal neural regulation, impaired gut motility, microbial dysbiosis, and barrier disruption, they may gradually develop into systemic inflammatory and metabolic abnormalities, creating conditions for gut-derived signals to influence local spinal cord inflammatory responses. Although the diversity, composition, and metabolic functions of gut microbes change after SCI, stable and reproducible microbial signatures have yet to be established, which may be due to differences in injury severity, injury completeness, disease stage, diet, antibiotic exposure, hospital environment, and detection methods (Zhang et al., 2018; Gur Arie et al., 2024; Pagán-Rivera et al., 2025). Therefore, the focus should shift from microbial classification or abundance to the functional consequences of dysbiosis, including barrier injury, LPS overflow, reduced SCFAs, altered tryptophan metabolism, and broader metabolic remodeling.

The intestinal barrier consists of epithelial cells, tight junctions, a mucus layer, and the mucosal immune system, which together prevent luminal microbes and metabolites from entering the circulatory system (Suzuki, 2020). After SCI, reduced gut motility, local hypoperfusion, microbial dysbiosis, and mucosal immune disturbance may converge to disrupt barrier homeostasis, reduce tight-junction protein expression, increase intestinal permeability, and allow gut-derived bacteria and metabolites to enter the peripheral circulation (Chen et al., 2025). In the gut-spinal cord axis, microbial dysbiosis and intestinal barrier injury mutually reinforce each other. Reduced motility and luminal disturbances directly disrupt the gut microbial ecosystem, while dysbiotic microbiota and altered microbial metabolism further impair epithelial barrier and mucosal immune homeostasis (Jing et al., 2023; Kigerl et al., 2018; Bazzocchi et al., 2021). LPS overflow, reduced protective metabolites such as SCFAs, and altered tryptophan metabolism are typical manifestations of microbiota-barrier imbalance. During the progression of SCI, these changes may lead to peripheral immune imbalance, abnormal activation of molecular pathways, and altered glial cell stress responses, thereby promoting secondary SCI (Zhu et al., 2024; Pagan-Rivera et al., 2024).

Overall, the key feature of intestinal changes after SCI is that the combined damage to microbial function and barrier integrity alters the way the gut releases inflammatory and metabolic signals to the periphery. Gut dysmotility and changes in the luminal environment provide upstream conditions for microbial dysbiosis; microbial dysbiosis and mucosal immune disturbance further weaken barrier integrity; and barrier disruption promotes LPS overflow and a reduction in protective metabolites, such as SCFAs, leading to a shift in peripheral immunity toward a pro-inflammatory state. Therefore, the intestinal changes after SCI may provide a basis for gut-derived signals to influence local spinal cord inflammation through the intermediate chain of microbial functional disturbance, barrier disruption, and peripheral immune imbalance.

## 3 Gut microbiota-derived inflammatory and metabolic signals after SCI

Once intestinal homeostasis is disrupted after SCI, microbial inflammatory molecules and metabolites can act as important mediators linking intestinal disturbance to secondary neuroinflammation. Focusing on functional changes and specific signals released or depleted is more helpful in explaining how the gut-spinal cord axis participates in the pathology of SCI compared with a purely compositional description of the microbiota. In current studies, LPS overflow mainly represents an increase in pro-inflammatory input following barrier disruption; a reduction in SCFAs indicates a loss of protective microbial metabolic function; alterations in tryptophan metabolism reflect a disruption in host-microbe metabolic interactions; while TMAO, bile acids, and other emerging metabolites indicate a broader metabolic-inflammatory remodeling after SCI. The following sections summarize these signals by category, focusing on their potential action points and the strength of evidence.

### 3.1 LPS and leakage of peripheral inflammatory signals

In the gut-spinal cord axis, LPS is a representative gut-derived pro-inflammatory signal that leaks out after intestinal barrier disruption. Under normal circumstances, LPS is primarily confined within the gut lumen by an intact epithelial barrier, mucus layer, and mucosal immune system. After SCI, autonomic dysfunction, reduced gut motility, luminal stasis, microbial dysbiosis, and mucosal barrier injury can increase intestinal permeability, promoting the transfer of LPS into the peripheral circulation. Therefore, increased LPS in circulation can be viewed as an indicator of impaired intestinal barrier function and enhanced gut-derived pro-inflammatory input. In terms of evidence, LPS-related research is relatively concentrated in the field of the SCI gut-spinal cord axis. Animal experiments and some clinical observations suggest that SCI is associated with intestinal homeostatic disturbance, barrier decline, bacterial translocation, and systemic pro-inflammatory states, which are related to the disruption of the BSCB and changes in the inflammatory background of injured tissues (Rong et al., 2021; Li et al., 2022; Myers et al., 2019; Diaz et al., 2021). In chronic SCI, long-term bowel dysfunction, dysbiosis, and low-grade systemic inflammation may collectively sustain gut-derived pro-inflammatory input, making LPS overflow a component of the chronic inflammatory state after SCI. These findings support the biological rationale of the intestinal barrier injury-LPS overflow-peripheral inflammation axis after SCI.

However, it is important to note that current evidence mainly shows associations between LPS overflow, systemic inflammation, barrier damage, and the inflammatory background of lesions. It has not yet been proven that gut-derived LPS reaches the injured spinal cord in effective concentrations and directly acts on specific glial subpopulations. Thus, LPS is more appropriately defined as an upstream pro-inflammatory input that enters circulation after barrier failure, rather than a confirmed cell-specific inflammatory driver. Future research should combine gut-derived LPS tracking, dynamic permeability assays, receptor blockade, and cell-type-resolved analyses to clarify its source, trans-barrier transport, local exposure levels, and potential target cells.

### 3.2 Reduced SCFAs and imbalance of anti-inflammatory homeostasis

Short-chain fatty acids, mainly acetate, propionate, and butyrate, are the main metabolites produced by gut microbiota fermenting dietary fibers. Unlike LPS, which represents pro-inflammatory input, SCFAs reflect microbial metabolic activity and serve as a regulatory link between the gut ecosystem, peripheral immune balance, and inflammatory responses in the injured spinal cord (Liu et al., 2023). Under physiological conditions, SCFAs support the integrity of the epithelial barrier, maintain mucosal immune balance, and modulate peripheral immune-cell function to prevent excessive inflammatory stress. After SCI, the reduction of SCFA-producing bacteria weakens protective microbial metabolism, decreases support for the intestinal barrier, impairs peripheral immune tolerance, and reduces endogenous anti-inflammatory regulation (Jing et al., 2023; Kong et al., 2023).

Different SCFAs may have functionally distinct roles in the gut-spinal cord axis. Acetate primarily participates in energy metabolism, maintaining microbial ecology and peripheral immune homeostasis. Propionate can influence T-cell differentiation and cytokine secretion through receptors such as GPR41 and GPR43, thereby supporting systemic immune balance. Butyrate is particularly important for barrier protection: it provides energy to colonocytes, promotes the expression of tight-junction proteins, and enhances epithelial integrity. It can also inhibit histone deacetylase activity, reshape the transcriptional program of inflammatory genes, suppress excessive NLRP3 inflammasome activation, and modulate pyroptotic responses (Chang et al., 2014; Macia et al., 2015).

Studies have shown that SCI can disrupt the gut microbial composition and reduce the metabolic activity of SCFA-producing bacteria, leading to decreased levels of SCFAs, weakened barrier maintenance, mucosal immune homeostasis, and peripheral anti-inflammatory regulation (Liu et al., 2023; Cui et al., 2025). Thus, the reduction of SCFAs after SCI not only reflects impaired microbial metabolism but also indicates decreased protective metabolic input and weakened anti-inflammatory homeostasis. Existing evidence supports the role of SCFAs, particularly butyrate, in barrier protection and immune regulation. However, their effects on local inflammation in the injured spinal cord may be mediated more through intestinal barrier function, peripheral immunity, and barrier-related changes rather than through a single known molecular pathway or specific cellular effects. It remains unclear whether acetate, propionate, and butyrate have similar effects at different stages of SCI; whether fecal, serum, cerebrospinal fluid, and spinal cord SCFA levels are consistently related; and whether SCFAs directly or indirectly influence local inflammation. Targeted metabolomics, dynamic sampling from multiple biological compartments, receptor blockade, and cell-type-resolved analysis are needed to determine the true contributions of specific SCFA subtypes to the gut-spinal cord axis after SCI.

### 3.3 Tryptophan metabolites and AhR signaling

Tryptophan metabolism is an important component of host-microbe metabolic interaction. Gut microbes can convert tryptophan into various indole derivatives, some of which act as endogenous ligands for AhR, participating in barrier maintenance, mucosal immune regulation, and inflammation control (Rothhammer et al., 2016; Cen et al., 2026). After SCI, changes in microbial composition and metabolic function may disrupt the tryptophan metabolic profile, reducing metabolites with barrier-protective or immunoregulatory properties, or altering the balance among metabolic branches. Compared with LPS and SCFAs, tryptophan metabolites better reflect the complexity of the microbial metabolic network and its interaction with host immune regulation.

Currently, direct evidence regarding the involvement of the tryptophan metabolite-AhR axis in the gut-spinal cord axis after SCI remains limited. A recent study using an SCI model reported that *Limosilactobacillus reuteri* DSM17938 may regulate tryptophan metabolism, activate AhR-related signaling, improve intestinal barrier integrity, and reduce pro-inflammatory microglial activation (Cen et al., 2026). This study provides an important clue, suggesting that tryptophan metabolites may be involved in inflammatory regulation after SCI, but it remains a single primary study, insufficient to define the tryptophan metabolite-AhR-glial response axis as an established pathological pathway in SCI. Other evidence regarding AhR’s regulation of barrier function, immune response, and CNS inflammation primarily comes from models of other CNS diseases, intestinal inflammation models, or *in vitro* studies.

Accordingly, the tryptophan metabolite-AhR axis should be viewed as a potential regulatory node connecting microbial metabolic disturbance, changes in intestinal barrier, and peripheral immune imbalance, rather than a fully validated mechanism of SCI. The direction and magnitude of its effects may depend on the identity of the ligands, disease stage, integrity of the BSCB, target cell types, and local inflammatory environment. Future research should define the sources, concentrations, trans-barrier transport capacity, and functional associations of key tryptophan metabolites after SCI, and verify whether they truly constitute key mechanisms in the gut-spinal cord axis through independent replication, dynamic metabolomics, and cell-type-resolved analyses.

### 3.4 TMAO and other emerging metabolites

In addition to LPS, SCFAs, and tryptophan metabolites, emerging signals such as TMAO, bile acids, succinate, lactate, and polyamines are receiving increasing attention. Not all of these metabolites have sufficient direct evidence related to SCI, but they suggest that gut dysbiosis after SCI may involve a broader metabolic-inflammatory remodeling. Compared with LPS and SCFAs, the evidence base for this group of signals is weaker and should be viewed as mechanistic clues and future research directions. TMAO is generated from microbial metabolism of substrates such as choline and carnitine, followed by hepatic oxidation, and is typically associated with metabolic inflammation, oxidative stress, and vascular injury. A recent preliminary study on SCI suggests that TMAO may exacerbate neuroinflammation after SCI and may involve the activation of oxidative stress and inflammasomes (Mcmillin and Demorrow, 2016; Butcher and Arthur, 2025). However, the current TMAO-NLRP3-microglia axis still primarily relies on one SCI study and should not be considered an established pathological pathway. Its temporal profile, dose–response relationship, main target cells, and their relative contributions to secondary neuroinflammation after SCI require further validation.

Bile acids are important metabolic signals that connect the gut microbiota, hepatic metabolism, and immune-inflammatory responses. Gut microbes convert primary bile acids into secondary bile acids, which can regulate metabolism, barrier function, and immune inflammation through receptors such as FXR and TGR5. Previous studies have indicated that abnormal bile acid metabolism in several neurological diseases may be associated with barrier injury, CNS inflammation, and glial cell activation, making bile acid signaling a potential direction for gut-spinal cord axis research (Butcher and Arthur, 2025). However, there is currently no direct evidence that bile acid dysmetabolism regulates BSCB injury, glial cell response, or secondary neuroinflammation after SCI through FXR/TGR5 or related pathways. Therefore, bile acid-related mechanisms should be viewed as extrapolated mechanistic clues rather than as validated SCI pathways. Succinate, lactate, and polyamines may also be involved in immunometabolic regulation, oxidative stress, and inflammation, but direct evidence in the gut-spinal cord axis of SCI is even more limited. Dynamic metabolomics, microbial functional analysis, barrier assessment, and functional blockade will be necessary to identify metabolites truly linked to neuroinflammatory progression after SCI, rather than indiscriminately incorporating all metabolic changes into the gut-spinal cord axis framework.

Taken together, LPS and SCFAs currently represent two categories of gut-derived signals with relatively concentrated evidence in the study of the gut-spinal cord axis in SCI. LPS primarily reflects enhanced pro-inflammatory input after intestinal barrier disruption, while the reduction of SCFA indicates a loss of protective microbial metabolic function. In contrast, the tryptophan metabolite-AhR axis, TMAO-NLRP3 axis, bile acid-FXR/TGR5 axis, and other emerging metabolites have mechanistic value, but most are still in early or extrapolated stages. Therefore, in this review, these signals are regarded as hypotheses that require further validation rather than established SCI pathways. Their downstream pathways, cellular targets, and relationships with functional outcomes should be clarified through dynamic metabolomics, barrier assessments, cell-specific analyses, and functional blockade.

To reduce subjectivity in the evidence stratification, we predefined the levels of evidence used in Table 1. Relatively concentrated SCI evidence refers to mechanisms supported by two or more studies related to SCI, preferably including *in vivo* studies and indicators of barrier integrity, peripheral immunity, BSCB status, or cellular responses. Preliminary SCI support refers to SCI models or interventions that indicate an association but are insufficient due to incomplete causal chains or cell-specific validation. Limited direct SCI evidence refers to mechanisms mainly supported by a single major SCI study. Extrapolated evidence refers to findings mainly derived from other CNS diseases, intestinal inflammation, metabolic inflammation, or *in vitro* models. On this basis, Table 1 specifies the sources of evidence, main types of studies, potential target cells, and remaining mechanistic gaps.

**Table 1:** Evidence stratification and mechanistic gaps for gut-derived signals mediating secondary neuroinflammation after SCI.

Mechanistic axis	Evidence type	Evidence tier	Potential target cells	Main process	Mechanistic gaps
LPS-TLR4/NF-κB	SCI animal studies, some clinical samples, and *in vitro* studies	Relatively concentrated SCI evidence	Peripheral monocytes/macrophages, microglia, etc.	LPS overflow initiates TLR4/NF-κB-dependent pro-inflammatory transcription; associated with BSCB injury	Lack of LPS tracing and cell-specific blockade; difficult to distinguish from DAMP contributions
SCFAs-Treg/Th17-NLRP3	SCI animal studies, SCFA/butyrate interventions, and immunometabolism studies	Preliminary SCI support	Treg/Th17 cells, myeloid cells, intestinal epithelium, and BSCB-related cells	SCFAs maintain barrier function and immune tolerance; butyrate may restrain excessive NLRP3 activation	Roles of different subtypes, disease stages, and cross-sample correspondence remain unclear
Tryptophan metabolites-AhR	Single SCI study plus extrapolation from other CNS disease and gut immunity studies	Limited direct SCI evidence	Intestinal epithelium and peripheral immune cells; potential glial/NVU-related cells	Indole metabolites activate AhR and participate in barrier and immune regulation	Key ligands, local target cells, and causal chain require validation
TMAO-NLRP3	Single SCI animal study plus extrapolation from metabolic inflammation and in vitro studies	Limited direct SCI evidence	Mainly myeloid cells; potential effects on endothelial cells and astrocytes	TMAO may enhance oxidative stress and promote NLRP3 activation	Lack of replication, dose-time analysis, and cell-specific evidence
Bile acids-FXR/TGR5	Other CNS disease, metabolic inflammation, and in vitro studies	Mainly extrapolated evidence	Intestinal epithelium, immune/endothelial cells, and potential NVU cells	FXR/TGR5 may regulate barrier function and immune-inflammatory responses	Direct SCI evidence is insufficient; key bile acid species and receptor dependence remain unclear
Other emerging metabolites (succinate, lactate, polyamines, etc.)	Metabolic inflammation and other CNS disease studies	Mainly extrapolated evidence	Peripheral immune cells and cells involved in metabolic reprogramming	May affect immunometabolism, oxidative stress, and inflammatory factor expression	Lack of dynamic SCI metabolomics and functional blockade evidence

LPS-TLR4/NF-κB and SCFAs-Treg/Th17-NLRP3 are the relatively well-supported mechanistic axes in SCI gut-spinal cord axis research, although their target cells and causal relationships remain to be clarified. Tryptophan metabolite-AhR, TMAO-NLRP3, bile acid-FXR/TGR5, and other emerging metabolites are valuable research directions, but some evidence is still extrapolated from other CNS diseases or metabolic inflammation and should not be treated as confirmed SCI mechanisms.

## 4 Key molecular pathways linking gut-derived signals to spinal neuroinflammation

After gut-derived signals enter the peripheral circulation, whether they can affect inflammation in the injured spinal cord depends on whether local cells can recognize them and convert them into sustained cellular responses through relevant molecular pathways. TLR4/NF-κB, NLRP3 inflammasome, and AhR represent pro-inflammatory recognition, inflammasome assembly, and metabolic-immune regulation, respectively. These pathways are important nodes for understanding how gut-derived signals convert into local spinal cord inflammation.

### 4.1 TLR4/NF-κB signaling mediates the amplification of inflammatory cascades

Among gut-derived inflammatory signals, LPS is a typical pro-inflammatory input, and the TLR4/NF-κB pathway is one of the core downstream pathways that receive LPS-mediated inflammatory stimuli and initiate the inflammatory response. After SCI, intestinal barrier impairment allows LPS, peptidoglycan, and other microbial-associated molecular patterns to enter circulation. Circulating LPS can be recognized by monocytes, macrophages, and other immune cells, initiating MyD88-dependent signaling through TLR4 and promoting NF-κB nuclear translocation. This process induces high expression of TNF-α, IL-1β, IL-6, CCL2, and other inflammatory mediators, amplifying systemic inflammatory stress. During the recovery process after SCI, inflammatory stimuli also include endogenous damage-associated molecular patterns released from the injured spinal cord tissue, such as HMGB1, ATP, and heat-shock proteins. These molecules can act on the TLR4/NF-κB and related inflammatory pathways, increasing gut-derived exogenous stimuli, thereby sustaining the inflammatory response at the lesion site and exacerbating local secondary injury. Therefore, gut-derived pro-inflammatory inputs and injury-derived stimuli may converge on the TLR4/NF-κB signaling pathway, jointly enhancing the stress response of microglia, infiltrating macrophages, and astrocytes (Fan et al., 2020; Liang et al., 2022). This convergence is an important reason why inflammation may persist and amplify after SCI.

The TLR4/NF-κB signaling pathway can also interact with MAPK, JAK/STAT, cGAS-STING, and other pathways to promote cytokine secretion, cellular stress, glial cell activation, and immune responses, collectively forming a complex inflammatory regulatory network after SCI (Guo et al., 2023; Huang et al., 2024; Fan et al., 2025). However, the TLR4/NF-κB signaling pathway exhibits significant temporal heterogeneity and may have different effects at different stages of injury. Moderate activation in the early post-injury phase may initiate the inflammatory response, clear necrotic tissue, and create conditions for subsequent repair; whereas sustained excessive activation may promote the release of pro-inflammatory mediators, delay the resolution of inflammation, and exacerbate secondary neuroinjury. Therefore, interventions targeting TLR4/NF-κB should not be simply understood as unidirectional inhibition but should be considered in conjunction with the injury stage, inflammatory activity, BSCB integrity, and local microenvironmental features.

### 4.2 NLRP3 inflammasome and pyroptosis

The NLRP3 inflammasome is a major molecular platform in secondary neuroinflammation after SCI. It can convert local injury signals, peripheral pro-inflammatory inputs, and metabolic disturbances into the maturation of cytokines and pyroptotic responses. After SCI, local factors such as the release of DAMPs, oxidative stress, mitochondrial dysfunction, and ionic imbalance provide the basis for NLRP3 activation. In the context of intestinal barrier impairment and enhanced peripheral inflammation, gut-derived pro-inflammatory inputs and metabolic abnormalities may further exacerbate this process. Mechanistically, the activation of the NLRP3 inflammasome typically involves two steps: priming and activation. The priming step is usually mediated by upstream signals such as TLR4/NF-κB, which increase the expression of NLRP3, pro-IL-1β, and pro-IL-18. The activation step is driven by oxidative stress, mitochondrial injury, and metabolic abnormalities, promoting the assembly of the NLRP3-ASC-pro-caspase-1 complex. Activated caspase-1 subsequently drives the maturation of IL-1β and IL-18 and mediates pyroptosis through GSDMD, thereby exacerbating inflammatory injury at the lesion site (Kelley et al., 2019; Wang et al., 2022).

Within the framework of the gut-spinal cord axis, the NLRP3 inflammasome can be viewed as a convergence platform for peripheral inflammatory inputs, microbial metabolic disturbances, and local injury signals. After intestinal barrier disruption, LPS overflow may provide a priming signal for NLRP3 activation through pro-inflammatory recognition; reduced SCFAs may weaken endogenous suppression of excessive inflammation; while pro-inflammatory metabolites such as TMAO may influence the activity of the inflammasome through oxidative or metabolic stress. Thus, gut-derived signals do not act on NLRP3 in the same manner but participate at different levels, including pro-inflammatory priming, loss of anti-inflammatory regulation, and enhancement of metabolic stress. The activation of NLRP3 is also influenced by local regulators within the injured spinal cord. NEK7 can promote the assembly of the NLRP3 inflammasome and enhance pro-inflammatory signaling in microglia/macrophages after SCI, whereas CD73 may limit microglial pyroptosis through the PI3K/AKT/Foxo1 axis. Some metabolites, including butyrate, β-hydroxybutyrate, and omega-3 polyunsaturated fatty acids, may partially inhibit excessive NLRP3 activation and reduce central inflammatory injury after SCI (Sun et al., 2016; Qian et al., 2026; Baazm et al., 2021). These findings indicate that the activation of NLRP3 is not driven by a single gut-derived signal but dynamically varies under the combined influence of local injury signals, peripheral inflammatory inputs, and metabolic regulation.

Importantly, there are differences in the level of evidence for the signal-NLRP3 connection. LPS-related priming has a clear inflammatory biological basis, while the inhibitory effect of SCFAs/butyrate on excessive NLRP3 activation has some experimental support. In contrast, the TMAO-NLRP3 axis in SCI still primarily relies on a single primary study and cannot yet be considered an established pathological pathway. The NLRP3 inflammasome is therefore better understood as a convergence platform for multiple inflammatory and metabolic stimuli rather than a linear pathway driven by a single gut-derived signal. Future research should integrate disease-stage stratification, inflammasome activity assays, cell-type-resolved analyses, and functional blockade to define the relative contributions of different gut-derived signals in the processes of NLRP3 priming, activation, and resolution.

### 4.3 Multi-pathway crosstalk

Secondary neuroinflammation after SCI is not driven by a single molecular pathway but develops as a network process involving pro-inflammatory recognition, inflammasome activation, metabolic-immune regulation, and barrier injury. The TLR4/NF-κB, NLRP3, and AhR pathways discussed above represent different levels of inflammatory regulation. TLR4/NF-κB primarily mediates the recognition of exogenous or endogenous pro-inflammatory signals and initiates the transcription of inflammatory genes; the NLRP3 inflammasome further drives the maturation of IL-1β and IL-18 and cell pyroptosis; while AhR-related signaling more closely reflects the regulatory relationship among microbial metabolites, barrier function, and immune homeostasis. These pathways are not isolated but interact within the complex inflammatory context after SCI.

In terms of pathway relationships, TLR4/NF-κB provides the transcriptional priming required for NLRP3 activation by increasing NLRP3, pro-IL-1β, and pro-IL-18. Oxidative stress, mitochondrial dysfunction, and metabolic abnormalities subsequently promote the assembly and activation of the inflammasome. At the same time, AhR-related signaling may indirectly regulate the intensity of the TLR4/NF-κB and NLRP3 inflammatory responses by affecting barrier homeostasis, immune tolerance, and metabolic inflammation. Therefore, the molecular signaling within the gut-spinal cord axis is not a simple linear transmission process, but rather an intertwined network involving pro-inflammatory recognition, inflammasome activation, and metabolic-immune regulation.

In addition, local injury signals after SCI can amplify this pathway crosstalk together with peripheral inflammatory inputs. DAMPs, oxidative stress products, and cell death signals released from the lesion can interact with peripheral inflammatory or metabolic signals, keeping TLR4/NF-κB, NLRP3, AhR, and related pathways in a dynamic state (Dong et al., 2025b; Anwar et al., 2016; Ji et al., 2021; Papatheodorou et al., 2017). In this process, BSCB injury is not only a gateway for peripheral signals to enter the CNS; it may also alter the sensitivity of the lesion to inflammatory and metabolic stimuli, thereby affecting the intensity, duration, and resolution of pathway activation.

**Figure 2:**
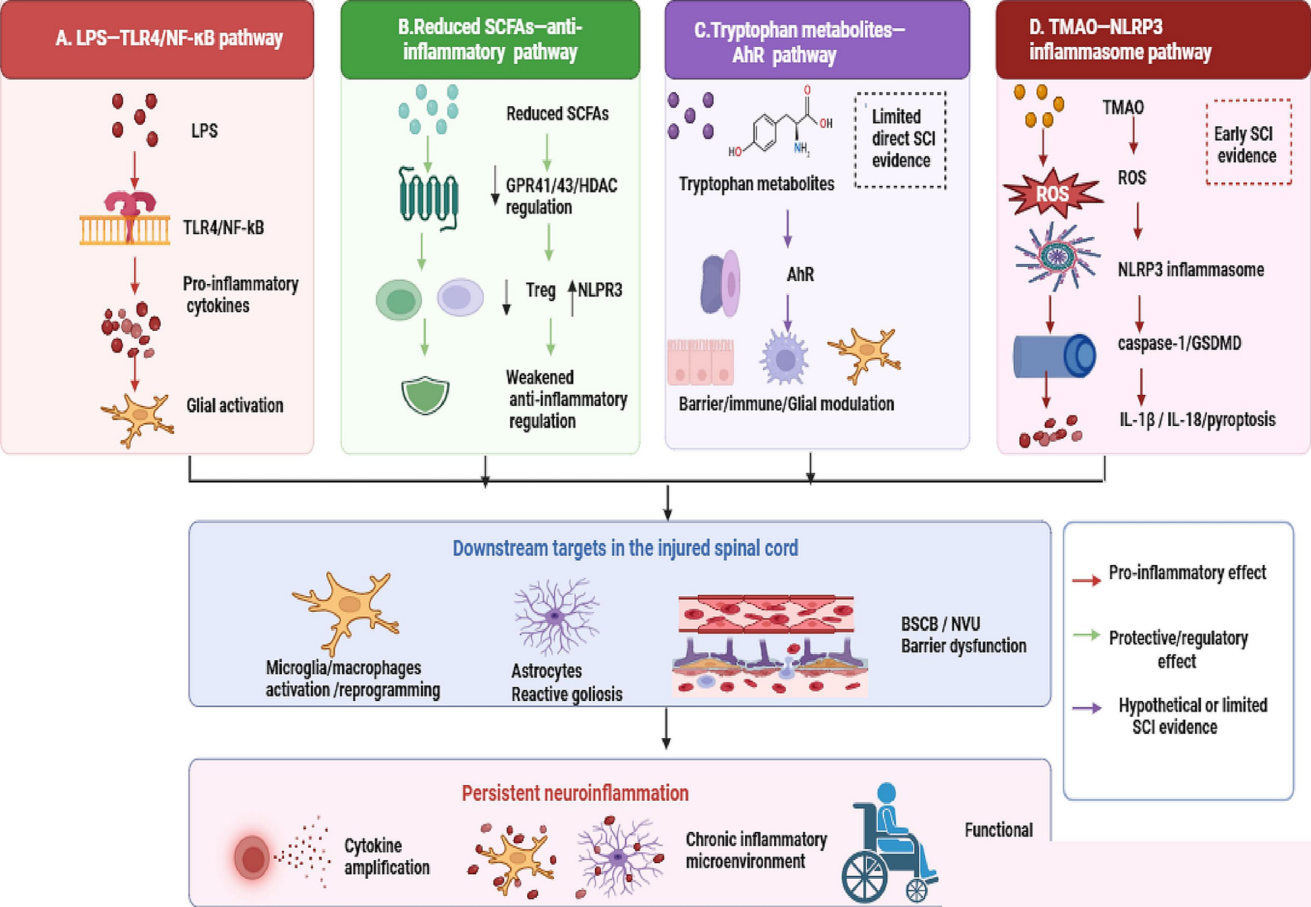
Molecular pathways by which gut-derived signals may modulate glial responses after SCI. LPS may activate TLR4/NF-κB and related inflammatory pathways; reduced SCFAs may weaken anti-inflammatory regulation and barrier protection; tryptophan metabolites may act through AhR-related signaling; and TMAO may promote oxidative and inflammasome-related stress. These pathways converge on myeloid cells, astrocytes, and the BSCB/NVU, contributing to persistent neuroinflammation.

Therefore, gut-derived signals involved in secondary neuroinflammation after SCI should not be interpreted as one signal corresponding to a fixed pathway. Instead, in the context of intestinal barrier disruption, peripheral inflammatory inputs, BSCB injury, and persistent local damage signals, multiple molecular pathways jointly participate in the initiation, amplification, maintenance, and resolution of inflammation. Future research should examine the dynamic roles of these pathways in acute, subacute, and chronic stages and utilize pathway blockade, metabolic intervention, and barrier assessment to determine their relative contributions to gut-spinal cord axis-mediated secondary neuroinflammation (Figure 2).

## 5 Major cellular effectors and network consequences of gut-derived signals

Once gut-derived signals enter the circulation, they do not independently determine the direction of neuroinflammation after SCI. Instead, they act through peripheral immune inputs, dual-barrier injury, and cellular crosstalk within the lesion. This section focuses on the main cellular effectors, including microglia, infiltrating macrophages, astrocytes, and the BSCB/NVU.

### 5.1 Microglia and infiltrating macrophages

Microglia and infiltrating macrophages are the main components of the myeloid-cell response in the injured spinal cord after SCI, but they differ in origin and function. Microglia originate from yolk-sac myeloid progenitors during embryogenesis and reside long-term in the CNS, participating in immune surveillance, synaptic regulation, and maintenance of microenvironmental homeostasis. After SCI, resident microglia are rapidly activated and migrate to the lesion core and surrounding areas, participating in the early inflammatory response and clearance of necrotic tissue. Infiltrating macrophages primarily originate from peripheral monocytes and enter the lesion after BSCB breakdown, participating in inflammatory amplification, debris clearance, and tissue remodeling. Circulating monocytes can infiltrate spinal cord lesions and, under the influence of the local inflammatory microenvironment, undergo phenotypic conversion into macrophage-like cells with specific functions (Greenhalgh et al., 2018; Kobayakawa et al., 2019). During the ongoing progression of SCI, activated microglia and infiltrating macrophages work together to dynamically remodel the inflammatory microenvironment and shape the trajectory of local inflammation.

From the perspective of the gut-spinal cord axis, gut-derived signals may first act on the peripheral immune system and then influence local inflammation through BSCB injury and immune-cell infiltration. After SCI, intestinal barrier injury allows LPS and other microbial-associated molecules to enter circulation, activating peripheral monocytes, macrophages, neutrophils, and related immune cells, and promoting the release of TNF-*α*, IL-1β, IL-6, CCL2, and other mediators through pathways such as TLR4/NF-κB (Liu et al., 2017). As BSCB permeability increases, peripheral cytokines, activated immune cells, and some abnormal gut-derived metabolic signals more easily enter or affect the injured spinal cord, thereby altering the responses of microglia and infiltrating macrophages. Thus, the influence of gut-derived signals on lesion myeloid cells may not only be a direct stimulation of resident microglia in the CNS but may more likely involve a continuous process of intestinal barrier disruption, peripheral myeloid-cell activation, increased BSCB permeability, and remodeling of myeloid-cell responses within the lesion.

At the specific signaling level, pro-inflammatory metabolites such as TMAO may enhance oxidative stress, activate the NLRP3 inflammasome, promote the release of IL-1β and IL-18, and induce pyroptosis, thereby sustaining the pro-inflammatory state of microglia and infiltrating macrophages. In contrast, SCFAs, particularly butyrate, may limit excessive myeloid-cell inflammation by inhibiting HDAC, maintaining Treg function, and suppressing the NLRP3 inflammasome. Tryptophan metabolites may also influence the immunoregulatory state of myeloid cells through AhR signaling. Therefore, gut-derived signals are not merely enhancing or suppressing inflammation. They may jointly determine whether lesion inflammation is persistent or resolves by modulating peripheral immune-cell activation, BSCB permeability, local myeloid transcriptional programs, and inflammasome activity. In current SCI research, microglia and infiltrating macrophages are often analyzed together, making it difficult to determine whether gut-derived signals primarily act on resident microglia or peripheral macrophages. This issue needs to be further validated through cell-type-resolved and functional blockade approaches.

### 5.2 Reactive astrocytes

Astrocytes are important cellular participants in inflammatory microenvironment remodeling, lesion-boundary formation, and glial scar construction after SCI. Their responses are bidirectional. Moderate activation has a protective effect, stabilizing the tissue structure of the injured area, limiting the spread of the inflammatory lesion into adjacent tissues, and reducing extensive secondary injury. If activation is sustained or excessive, astrocytes may release pro-inflammatory cytokines and chemokines and produce inhibitory extracellular-matrix components, thereby maintaining chronic inflammation and limiting axonal regeneration (Zhou Z. L. et al., 2023; Ageeva et al., 2024). Therefore, astrocytes after SCI should not be viewed solely as structural scar-forming cells; they have context-dependent effects on inflammation control, tissue isolation, repair initiation, and regeneration limitation.

Currently, evidence regarding the direct action of gut-derived signals on astrocytes remains limited. Based on the mechanisms mentioned above, LPS overflow, reduced SCFAs, and altered tryptophan metabolite-AhR signaling are more likely to first change the local spinal microenvironment through peripheral immune imbalance, increased BSCB permeability, and myeloid-cell activation, thereby providing upstream conditions for the reactivity of astrocytes. Microglia and infiltrating macrophages may serve as important intermediaries linking gut-derived signals to astrocyte responses. Gut-derived LPS can enhance pro-inflammatory myeloid activity through TLR4/NF-κB and related pathways, promoting the release of factors such as TNF-*α*, IL-1β, and CCL2. Activated microglia can also produce IL-1a, TNF-α, and C1q, which drive astrocytes toward pro-inflammatory or neurotoxic-related reactive states. Reactive astrocytes can subsequently release chemokines, complement-related molecules, and extracellular-matrix components, further influencing immune-cell recruitment, microglial states, and the inflammatory properties of glial scars. Thus, gut-derived pro-inflammatory inputs may not directly drive the activation of astrocytes but may first alter peripheral myeloid cells and lesion microglia/macrophages, and then induce functional remodeling of astrocytes through cytokines, chemokines, and complement-related signals.

Once formed, reactive astrocytes further participate in the maintenance of injury-induced inflammation and spatial regulation. They can upregulate complement-related molecules such as C3, secrete chemokines like CCL2 and CXCL10, and alter the composition of the extracellular matrix, thereby affecting the recruitment of peripheral immune cells, activation of microglia, and the inflammatory properties of glial scars (Qiao et al., 2022; Zhao et al., 2023; Pineau et al., 2010). Astrocytes are also closely related to the BSCB/NVU. Their perivascular end-feet help regulate the homeostasis of endothelial cells, pericytes, and tight junctions. When gut-derived inflammatory inputs persist and exacerbate BSCB injury, astrocytes become both recipients of inflammatory stimuli and regulators of the entry of peripheral immune cells into the lesion through barrier-related signaling and extracellular matrix deposition.

Overall, astrocytes are more likely to be indirectly affected after gut-derived signals pass through peripheral immunity, BSCB injury, and myeloid-cell responses. Direct evidence of SCI shows that gut-derived signals driving astrocytic phenotypic conversion remain insufficient. Future series of studies should determine their roles in pro-inflammatory signaling, barrier regulation, and glial scar biology.

### 5.3 Neurovascular unit and BSCB

The BSCB and its associated neurovascular unit form a critical structural interface that connects peripheral gut-derived inflammatory and metabolic signals with the local microenvironment of the injured spinal cord. After SCI, endothelial injury, tight-junction degradation, pericyte dysfunction, and remodeling of astrocytic end-feet within the neurovascular unit collectively compromise the integrity of the BSCB and significantly increase permeability. Once the barrier function is impaired, LPS, inflammatory mediators, immune cells, and some gut microbiota-derived metabolites, which are typically restricted to the periphery, can more easily enter or affect the injured spinal cord (Zhou et al., 2023a; Zhou et al., 2023b). Thus, the increased permeability of the BSCB not only allows peripheral signals to enter the CNS to a greater extent but may also further remodel the inflammatory microenvironment of the lesion, leading to secondary inflammatory injury after SCI.

In the sequential process of the gut-spinal cord axis, the intestinal barrier and BSCB constitute a connected double-barrier system. Intestinal barrier injury after SCI permits LPS, abnormal metabolites, and inflammatory mediators to enter the peripheral circulation. If BSCB/NVU injury occurs simultaneously, these peripheral signals can more easily reach or affect the microenvironment of the injured spinal cord. Double-barrier injury is therefore an important entry point for understanding how gut-derived signals influence local inflammation after SCI. Different gut-derived signals may affect the BSCB in different ways. Pro-inflammatory signals such as LPS can activate peripheral immunity, potentially exacerbate endothelial injury, promote immune-cell infiltration, and enhance glial cell responses, thereby further expanding BSCB dysfunction. According to evidence from other CNS diseases and barrier studies, SCFAs and indole-derived tryptophan metabolites may have barrier-protective effects, but whether they directly maintain the homeostasis of the BSCB/NVU after SCI remains to be verified (Xie et al., 2022; Chenghan et al., 2025). Thus, the BSCB/NVU is not only a structural passage for peripheral signals to enter the CNS but also a potential interface for gut-derived signals to regulate the inflammatory response at the lesion.

Accordingly, the neurovascular unit should not be viewed merely as a passive barrier blocking peripheral signals. After SCI, the disruption of the BSCB increases the opportunity for peripheral inflammatory signals to affect the lesion, while dysfunction of endothelial cells, pericytes, and astrocytic end-feet can alter the extent of inflammatory spread and the degree of immune-cell entry. In other words, the BSCB/NVU serves as both an entry point for gut-derived signals to influence the microenvironment of the injured spinal cord and an important node for regulating immune-cell infiltration, glial responses, and inflammatory diffusion. Future research should clarify whether intestinal barrier injury and BSCB disruption mutually reinforce each other and distinguish the effects of different gut-derived signals on various NVU cell components. This will help define the mechanistic significance of double-barrier injury in secondary neuroinflammation after SCI.

### 5.4 Immune cell–glial crosstalk and its spatiotemporal heterogeneity

The influence of gut-derived signals on the inflammatory microenvironment after SCI is typically not achieved by directly acting on a specific type of glial cell, but rather through gradual interactions between peripheral immune cells and lesion glia. After SCI, intestinal barrier disruption and altered microbial metabolism can impair peripheral immune homeostasis and change the composition, activation state, and cytokine expression of immune cells entering the lesion. Under normal circumstances, SCFAs promote Treg differentiation and help maintain their immunosuppressive function, thereby limiting excessive inflammation. When SCFAs decrease after SCI, Treg-mediated anti-inflammatory regulation may weaken, and peripheral immunity may shift toward a pro-inflammatory state. In the context of BSCB disruption, enhanced peripheral cytokines and immune-cell activation signals can more easily affect the local spinal cord microenvironment. Treg cells may also modulate microglial cell states through STAT3 and related pathways, influencing the intensity of inflammation and tissue repair. This suggests that the effects of gut-derived metabolites on glia may not be due to direct stimulation, but rather involve changes in peripheral immune-cell function first, followed by indirect regulation of microglia, infiltrating macrophages, and astrocytes through immune-cell infiltration or soluble mediators.

Glial cells in the lesion of SCI do not participate in pathological responses in isolation. Instead, they shape local inflammation through continuous intercellular interactions. Activated microglia can secrete TNF-α, IL-1β, and other inflammatory mediators and activate complement-related signals. These pathological signals are important triggers for the functional remodeling of astrocytes and can drive homeostatic astrocytes to transition to a reactive phenotype. Reactive astrocytes can subsequently secrete chemokines, alter extracellular matrix composition, and participate in barrier-related signaling, thereby affecting the quantity and spatial distribution of immune cells in the lesion. These changes, in turn, can regulate microglial activation, forming a mutually reinforcing inflammatory network between immune cells and glia.

The endothelial cells, pericytes, and astrocytic end-feet in the neurovascular unit are also involved in the formation and maintenance of this inflammatory network. Increased BSCB permeability after SCI allows peripheral cytokines, immune cells, and gut-derived pro-inflammatory signals to enter or affect the injured CNS more readily. Conversely, dysfunction of local glia and vascular-associated cells may expand the range of inflammatory signal propagation, promote immune-cell recruitment, and exacerbate secondary pathology within the lesion. Thus, local inflammation after SCI is not driven by a single cell type or pathway, but is a network process involving myeloid cells, astrocytes, and the neurovascular unit.

This network is not static across disease stages or lesion areas. The acute phase of SCI is characterized by BSCB disruption, DAMP release, LPS overflow, and rapid myeloid cell activation. The subacute phase involves inflammation resolution, tissue remodeling, BSCB repair, and glial scar formation. In the chronic phase, persistent microbial dysbiosis and low-grade systemic inflammation may lead to chronic activation of microglia and astrocytes (Qian et al., 2025). Spatially, there are differences in cell composition, barrier status, and inflammatory background among the lesion core, lesion border, and remote segments. Therefore, the effects of gut-derived signals on glia should not be simply classified as pro-inflammatory or anti-inflammatory. They should be assessed based on disease stage, lesion region, and local microenvironment.

## 6 Therapeutic implications and current limitations

Based on the above analysis, interventions targeting the gut-spinal cord axis should not be broadly described as microbiota regulation or general anti-inflammation. They should be evaluated along a continuous chain that connects microbial metabolism, intestinal barrier and peripheral immunity, BSCB, glial response, and functional outcomes. Ideally, evidence should indicate that an intervention not only improves microbial composition or metabolic function but also restores intestinal barrier and peripheral immune homeostasis, reduces gut-derived pro-inflammatory inputs or restores protective metabolites, alleviates BSCB injury and abnormal glial responses, and ultimately promotes neurological recovery (Table 2).

**Table 2:** Evidence significance of therapeutic strategies targeting the gut-spinal cord axis.

No.	Study	Intervention/study type	Main findings	Interpretive value
1	He et al. (2022), Pharmacological Research (Wang et al., 2022)	Resveratrol; SCI animal experiment	Improved gut microbiota and metabolites after SCI, suppressed microglial activation, and promoted motor recovery.	Preliminary SCI support: suggests a possible link among natural products, gut microbiota, metabolites, and microglial inflammation, but causal blockade is still needed.
2	Xue et al. (2025), Int J Biol Macromol (Papatheodorou et al., 2017)	Polygonatum polysaccharide; SCI animal experiment	Regulated gut microbiota and SCFA levels, inhibited microglial activation, and improved functional recovery.	Preliminary SCI support: suggests that herbal polysaccharides may influence neuroinflammation through the microbiota-SCFA axis, but key taxa, metabolites, and target cells require validation.
3	Cheng et al. (2022), Microb Biotechnol (Greenhalgh et al., 2018)	Electroacupuncture; SCI animal experiment	Regulated gut microecology and improved intestinal dysmotility in SCI rats.	Preliminary SCI support: supports acupuncture-related entry points through gut motility and microbiota, but continuous validation of BSCB and glial responses is lacking.
4	Zhou et al. (2024), Acupuncture Research (Kobayakawa et al., 2019)	Jiaji electroacupuncture; SCI animal experiment	Inhibited inflammation through the HMGB1/TLR4/NF-κB pathway.	Indirect SCI evidence: suggests that electroacupuncture can modulate inflammatory pathways, but does not fully prove the microbiota-BSCB-glia causal chain.
5	Su et al. (2023), Int J Pharm (Qian et al., 2026)	Curcumin nanomedicine; SCI animal experiment	Curcumin nanodelivery exerted anti-inflammatory and neuroregenerative effects.	Indirect evidence: supports anti-inflammatory and pro-repair effects of active herbal compounds, but gut microbiota and gut-derived metabolic mechanisms remain to be verified.
6	Liu et al. (2024), Front Pharmacol (Zhou Z. L. et al., 2023)	TCM component-loaded biomaterials; systematic review	Biomaterials carrying traditional Chinese medicine components show anti-inflammatory, antioxidant, and SCI repair-promoting potential.	Extrapolated evidence: supports future evaluation of TCM components plus biomaterials plus microbiota assessment, but gut-spinal cord axis validation in SCI models is still needed.

The grading in **t**able is not based on the magnitude of the treatment effect. It is based on whether the study provides continuous evidence linking microbiota or metabolite changes, intestinal barrier and peripheral immune indicators, BSCB status, glial responses, and functional outcomes. Studies lacking key links are treated here as preliminary, indirect, or extrapolated evidence.

### 6.1 Interventions directly supporting the gut–spinal cord axis mechanism

The first category of interventions is currently closest to the causal chain of the gut-spinal cord axis, mainly including fecal microbiota transplantation (FMT) and SCFA/butyrate supplementation. A common feature of these studies is that the interventions directly target the gut microbiota or microbiota-derived metabolites, allowing for the simultaneous observation of multiple steps, including intestinal barrier, peripheral immunity, local spinal cord inflammation, and functional recovery.

The FMT may improve dysbiosis and intestinal barrier injury after SCI by reshaping microbial composition and metabolic function, thereby reducing gut-derived pro-inflammatory inputs. Some animal studies have shown that FMT can reduce systemic inflammation, improve the local inflammatory environment of the injured spinal cord, and promote motor recovery (Jing et al., 2021; Jing et al., 2022). These findings suggest that microbial remodeling may be involved in the regulation of secondary injury after SCI. However, the effects of FMT cannot be equated with those of specific bacterial taxa or metabolites. Its effects may involve microbial structure, metabolites, intestinal barrier, peripheral immunity, and neuroendocrine regulation (Xi et al., 2024). Therefore, although FMT supports the involvement of the gut-spinal cord axis in SCI repair, key steps still need to be clarified through reverse validation in recipient animals, antibiotic depletion, supplementation of specific taxa, and metabolite blockade.

SCFA/butyrate supplementation is another representative intervention. SCFAs, particularly butyrate, help maintain the integrity of the epithelial barrier, promote immune tolerance, and may reduce inflammatory responses through HDAC inhibition, GPR41/43 signaling, and NLRP3 inflammasome regulation. Existing studies indicate that SCFA supplementation or butyrate-related interventions can improve the inflammatory status after SCI and are associated with neurological recovery (Jing et al., 2023). Compared with FMT, SCFAs/butyrate have relatively defined targets and can be more directly linked to the pathological chain of reduced microbial metabolites, impaired anti-inflammatory homeostasis, and enhanced glial inflammation. However, different SCFA subtypes may have varying effects during the acute, subacute, and chronic stages of SCI, and their dynamic relationships in feces, serum, cerebrospinal fluid, and spinal cord tissue still need to be validated.

Overall, FMT and SCFA/butyrate interventions support the concept that changes in the microbiota or metabolites are involved in secondary neuroinflammation after SCI. Whether they fully prove the causal chain of microbiota-metabolite-BSCB-glial response-functional recovery depends on future research, which will continue to assess changes in metabolites, BSCB status, local cellular responses, and functional outcomes.

### 6.2 Interventions with evidence for microbiota or metabolic modulation but insufficient glial mechanistic validation

The second category includes resveratrol, *Polygonatum* polysaccharides, probiotics, and dietary fibers. These interventions have some evidence of affecting microbial composition, microbial metabolites, or intestinal barrier function and may reduce systemic inflammation or improve functional recovery after SCI. However, most studies have not sufficiently demonstrated whether the protective effects primarily depend on the gut microbiota and its metabolites, nor have they continuously validated the status of the BSCB, local molecular pathways, and glial responses.

Resveratrol has antioxidant, anti-inflammatory, and metabolic regulatory properties. Some SCI animal studies suggest that resveratrol improves gut microbiota and metabolite changes, reduces microglial activation, and promotes motor recovery (He et al., 2022; Kan et al., 2023). This suggests that its effects may not be limited to direct anti-inflammatory effects but may also involve the connection between microbial metabolic regulation and the alleviation of neuroinflammation. Nevertheless, existing evidence mainly comes from animal experiments and cannot determine whether the key effects of resveratrol necessarily stem from microbiota changes or whether they are also influenced by its direct antioxidant, anti-inflammatory, and neuroprotective properties.

Herbal polysaccharides, such as Polygonatum polysaccharide, also show potential in regulating the gut microbiota. Related studies suggest that they may improve microbial composition, increase SCFA levels, inhibit microglial activation, and enhance motor function (Ren et al., 2024; Xue et al., 2025). Similar to resveratrol, these findings provide valuable clues for the herbal polysaccharide-microbiota-SCFAs-neuroinflammation axis, but key bacterial taxa, metabolites, and the direct effects on local spinal cord cellular responses still need further definition.

Probiotics and dietary fibers primarily exert their effects by improving the gut ecosystem, increasing protective metabolites, and maintaining barrier function. Theoretically, these interventions may reduce gut-derived pro-inflammatory inputs and influence peripheral immunity by increasing SCFAs and regulating the Treg/Th17 balance (Liu et al., 2022; Mazziotta et al., 2023). In SCI research, however, evidence remains largely limited to microbial changes, improved intestinal function, or reduced inflammatory markers, lacking sustained validation linking the BSCB, myeloid cells, astrocytes, and functional recovery. Therefore, probiotics and dietary fibers should be viewed more as potential interventions for the gut-spinal cord axis rather than strategies with clearly defined mechanisms.

### 6.3 Interventions with evidence for neuroinflammation modulation or functional improvement but an incomplete gut–spinal cord axis causal chain

The third category includes electroacupuncture, some traditional Chinese medicine formulas, and other anti-inflammatory or pro-repair strategies. These interventions have a certain research basis for improving gut motility, inhibiting inflammation, protecting neurons, or promoting functional recovery after SCI, but whether their effects primarily depend on the gut-spinal cord axis still lacks sufficient support.

Electroacupuncture has attracted attention for its effects on gastrointestinal dysfunction and neurological recovery after SCI. Some studies suggest that electroacupuncture may improve gut motility, regulate the gut microbiota, reduce inflammation, and affect inflammatory pathways such as TLR4/NF-κB and HMGB1. Mechanistically, electroacupuncture may act through neural regulation, immune modulation, and gut microecological regulation (Cheng et al., 2022; Zhou et al., 2024). Precisely because it acts at multiple levels, it remains difficult to determine whether its primary mechanism in improving neuroinflammation after SCI originates from changes in the gut microbiota and its metabolites. Without assessing the microbiota, metabolites, intestinal barrier, BSCB, and glial responses together, the evidence can only indicate that electroacupuncture is related to the gut-spinal cord axis, but cannot prove that it acts through this axis.

Some traditional Chinese medicine formulas, curcumin nanomedicines, hydrogels, and biomaterials can promote functional recovery after SCI by inhibiting oxidative stress, reducing inflammation, enhancing neuroregeneration, or improving the local microenvironment (Gu et al., 2023; Rybachuk et al., 2024; Liu et al., 2024). However, if a study does not assess the gut microbiota, intestinal barrier, and gut-derived metabolic signals, it should not be classified as evidence of the gut-spinal cord axis mechanism. A more appropriate statement is that these interventions have neuroinflammatory regulatory or functional benefits and should be further evaluated for their potential involvement in the gut-spinal cord axis, rather than being presented directly as gut-spinal cord axis interventions.

Accordingly, future intervention studies should shift from correlation to causal validation. They should simultaneously assess microbial composition, targeted metabolites, intestinal barrier, peripheral immunity, BSCB status, and local spinal cord cellular responses, combined with antibiotic consumption, reverse FMT, supplementation of specific taxa, metabolite supplementation, or metabolite blockade to define key mechanistic steps.

The importance of the evidence from intervention studies in Table 2 is graded based on whether they cover key causal links in the gut-spinal cord axis. Interventions that directly target the gut microbiota or microbiota-derived metabolites while simultaneously assessing intestinal barrier, peripheral immunity, BSCB, local glial responses, and functional outcomes are considered to provide relatively direct support for the gut-spinal cord axis mechanism. Studies that only show changes in microbial composition, metabolites, or intestinal barrier but lack validation of BSCB, glial, or functional blockade are classified as insufficient mechanistic validation. Studies mainly showing anti-inflammatory, antioxidant, pro-regenerative, or functional effects without assessing microbiota, gut-derived metabolites, and double-barrier changes are regarded as indirect or extrapolated evidence.

## 7 Discussion and future perspectives

This review argues that the abnormalities of the gut-spinal cord axis after SCI should not be understood merely as changes in microbial composition. Instead, they should be viewed as a continuous pathological process involving gut dysmotility, intestinal barrier disruption, abnormal gut-derived inflammatory and metabolic signals, BSCB injury, and imbalanced glial and neurovascular unit responses. Current evidence indicates that LPS overflow, reduced SCFAs, altered tryptophan metabolism, and increased TMAO may promote the onset and persistence of secondary neuroinflammation after SCI through TLR4/NF-κB, NLRP3 inflammasome, AhR signaling pathways, and immune-glial cell crosstalk. Therefore, future research should shift from questioning whether the microbiota changes to investigating how gut-derived signals convert into inflammatory consequences at the cellular and tissue levels within the injured spinal cord.

Several questions remain unresolved. First, it is currently unclear whether there are relatively stable and reproducible microbial signatures after SCI. Differences across studies may relate to injury level, injury completeness, disease stage, antibiotic exposure, diet, hospitalization status, and sample size. Therefore, future research should go beyond descriptions of increases or decreases in specific genera and focus more on microbial function and metabolic consequences. Second, the tryptophan metabolite-AhR axis and TMAO-NLRP3 axis currently rely mainly on single primary studies of SCI and require independent replication for validation. Whether AhR effects vary with ligand type, target cells, and disease stage, and whether TMAO is a key driver of inflammatory progression or merely a concomitant marker of metabolic disturbance, should not be concluded prematurely. Dose–response analyses, time-course studies, and cell-specific validations are needed to complete the causal chain.

Future work should not be limited to the analysis of single-layer microbial communities. There should be a gradual shift toward microbial function, metabolic features, and causal verification to determine whether these changes truly alter gut-derived signals and contribute to secondary neuroinflammation after SCI. Mechanistic studies can integrate metagenomics, targeted metabolomics, transcriptomics, and proteomics to identify key gut-derived signaling molecules and define downstream regulatory networks. For samples from different stages of SCI, longitudinal sampling and dynamic monitoring are recommended to compare metabolites in feces, serum, cerebrospinal fluid, and spinal cord tissue, thereby elucidating signal sources, transport patterns, and stage-specific characteristics. For cellular localization and causal validation, single-cell sequencing, spatial transcriptomics, lineage tracing, and cell-specific blockade should be used to determine whether gut-derived signals primarily act on resident microglia, infiltrating macrophages, astrocytes, or neurovascular unit-associated cells, and to distinguish stage- and region-specific effects. This focused research strategy avoids repeating the same methodological suggestions across various subsections and provides a clearer roadmap. From a translational perspective, the gut microbiota, metabolite levels, and inflammatory status of SCI patients are susceptible to the effects of antibiotic use, secondary infections, rehabilitation, and comorbidities. Therefore, a prospective SCI cohort should be established to systematically collect microbial profiles, metabolite levels, inflammatory markers, imaging data, and neurological outcomes while adequately adjusting for confounders. This work will allow for a more reliable assessment of the reproducibility of preclinical findings and help determine whether specific targets and interventions have true translational value.

Overall, the gut-spinal cord axis provides a promising research direction for interventions targeting secondary neuroinflammation after SCI. Future research, however, should go beyond broad statements about microbiota regulation or general anti-inflammatory effects. More mechanism-based, clinically translatable strategies should be developed based on clearer evidence tiers, causal relationships, and cell-specific mechanisms.
